# Genetic engineering of T cells for increased homing to the tumor site

**DOI:** 10.1186/2051-1426-2-S3-P19

**Published:** 2014-11-06

**Authors:** Manja Idorn, Gitte Holmen Olofsson, Hjalte List Larsen, Joost van den Berg, Özcan Met, Per thor Straten

**Affiliations:** 1 Center for Cancer Immune Therapy, Herlev, Denmark; 2 Danish Stem Cell Center (DanStem), København N, Denmark; 3 Center for Cancer Immunotherapy, Herlev, Denmark

## 

Adoptive cell transfer (ACT) using in vitro expanded T cells from biopsy material represents a highly promising treatment of disseminated cancer. ACT in its present form is rather crude and improvements seem within reach. Recruitment of transferred lymphocytes to the tumor site is a crucial step in ACT efficacy; however, quite few T cells actually reach the tumor site upon administration. In the present pre-clinical study we have genetically engineered T cells aiming at increasing the homing of T cells by matching expression of chemokine receptors on T cells to chemokines secreted by the tumor, thus improving anti-tumor efficacy of ACT. By PCR analysis we found that several malignant melanoma (MM) cell lines showed expression of cytokines CXCL8/IL-8, CXCL12/SDF-1 and CCL2, which was confirmed by ELISA analysis of MM conditioned medium. Taking advantage of mRNA electroporation we successfully transfected T cells with mRNA encoding the chemokine receptors CXCR2 or chimeric receptor CXCR4-R2 on the cell surface, of which the chimeric constructs contain the intracellular region of CXCR2 achieving a significant increase in cell surface expression on the T cell. Both the wildtype and chimeric chemokine receptors are functional *in vitro *and show an increase in Ca2^+ ^influx upon binding and mediate specific migration of receptor transfected T cells towards CXCL8 and CXCL12 respectively, as well as towards MM conditioned medium. Migration towards conditioned medium was abolished by the addition of neutralizing antibodies against the respective ligands. Using the NOG mouse model for xenograft assessment of migration in vivo of receptor transfected T cells showed that CXCR2 transfected T cells possess a slightly increased tumor infiltration compared to mock transfected, which seem to be "stuck" in the lungs. In conclusion, both our CXCR2 and CXCR4-R2 chimeric receptor is functional in vitro, and transfection with CXCR2 seemed to increase the homing of CXCR2 transfected cells to the tumor site, thus setting the stage for future clinical application.

